# An evaluation of the association between lockdown during the SARS-CoV-2 pandemic and prematurity at the Nice University Hospital

**DOI:** 10.3389/fped.2023.1201423

**Published:** 2023-06-02

**Authors:** Marine Lorenzi, Mathilde Mayerus, Sergio Eleni Dit Trolli, Amandine Hue-Bigé, Kévin Legueult, Isabelle Guellec-Renne, Bérengère François-Garret

**Affiliations:** ^1^ Department of Neonatology, Nice University Hospital, Nice, France; ^2^ Department of Clinical Research and Innovation (DRCI), Nice University Hospital, Nice, France

**Keywords:** SARS-CoV-2 pandemic, lockdown, prematurity, low weight at birth, stillbirth

## Abstract

**Aim:**

To study the association between lockdown in France due to the SARS-CoV-2 pandemic and premature births at the Nice University Hospital.

**Methods:**

Data concerning neonates born at the level III maternity of the Nice University Hospital and immediately hospitalised in the neonatal reanimation unit or the neonatology department of the hospital with their mothers between the 1st of January 2017 and the 31st of December 2020, included.

**Results:**

We did not find a significant decrease in the global number of premature births <37 weeks of gestation, in low weight at birth or a significant increase in stillbirths during lockdown compared to a period with no lockdown. The profiles of the mothers and their newborns were compared when birth occurred during lockdown vs. no lockdown.

**Conclusion:**

We did not find any evidence of an association between lockdown and prematurity at the Nice University Hospital. This result is in agreement with meta-analyses published in the medical literature. The possible decrease in factors of risk of prematurity during lockdown is controversial.

## 1. Introduction

Prematurity is a major public health problem. Premature births represent 75% of perinatal mortality and more than half of infantile morbidity over the long term. Between 50,000 and 60,000 infants are born prematurely each year in France (1).

There are many factors of risk of prematurity. They can be related to obstetrical elements, maternal history and environmental factors. Some of the factors of risk changed during lockdown due to the SARS-CoV-2 virus, in particular imposed inactivity, atmospheric changes, changes to daily living and an increase in hygiene. France went into lockdown as of Tuesday 17th of March 2020 up to Sunday the 10th of May 2020, included. All nurseries, schools, universities have been closed as well as restaurant and business not essential. Outdoor gatherings, family or friendly reunions were no longer allowed. Teleworking has been promoted and barrier measures have been put in place (wearing a mask, social distancing, hygine promotion) (2).

The main aim of our study was to examine the association between lockdown and the birth of premature newborns in the general population at the level III maternity of the University Hospital of Nice.

## 2. Materials et methods

### 2.1. Description of the study

An observational retrospective and monocentric study was performed at the neonatal reanimation unit and neonatal department of the University Hospital of Nice, a level III maternity.

The hospital's computer databases and written reports of biological results and hospitalisation were used to collect data.

### 2.2. Criteria of inclusion

**Figure 1:**
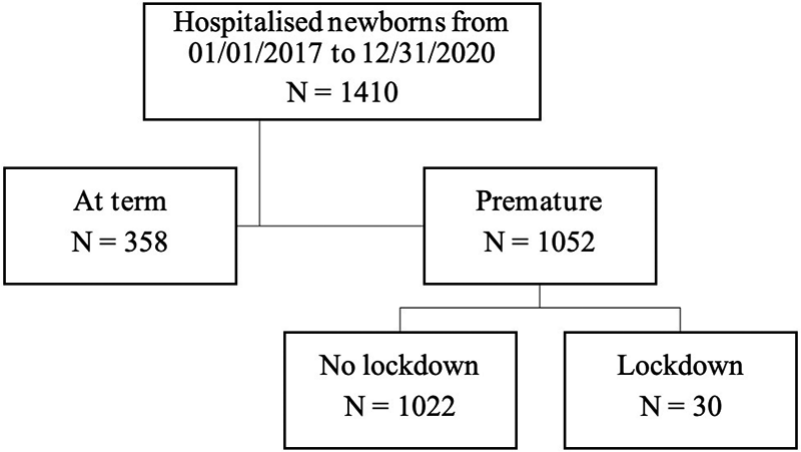
Flow chart.

All newborns including full-term infants born at the University Hospital of Nice immediately admitted into the neonatal reanimation unit or neonatal department between the 1st of January 2017 and 31st of December 2020 were included (Figure 1).

### 2.3. Data collection

The data included information concerning mothers and newborns. The information about the mother included: age, history of prematurity, injection of complete maturative antenatal corticosteroid therapy (two doses), antenatal administration of magnesium sulphate, mode of delivery (vaginal or caesarean) and the context of birth. The latter included risks of premature birth, premature rupture of membranes, vascular causes such as pre-eclampsia, metrorrhagia, suspicion of chorioamnionitis and other causes. The information about the newborn included: the weight and term of birth, the sex, administration of surfactant, the presence of broncho-pulmonary dysplasia defined as the need of supplementary oxygen for premature babies after 28 days of life, intra-ventricular hemorrage according to grade 3 (intra-ventricular hemorrage with dilation occupying more than 50% of the ventricule) and grade 4 Papile classification (intra-ventricular hemorrage with associated parenchymal lesions) (3), periventricular leucomalacia determined by lesions of the periventricular white matter, ulcero-necrotic enterocolitis characteristic of necrosis of the digestive wall classified according to the modified Bell score (stage 2a and 3b) (4), retinopathy of prematurity characterised by a proliferative disorder of the vessels of the retina, patent ductus arteriosus, microbial infection identified with a sample (blood or cerebrospinal fluid culture) and the occurrence or not of death.

### 2.4. Methods and statistical analysis

The total number of births and the term of the pregnancies were first analysed in a descriptive way from the 1st of January 2017 to the 31st of December 2020. The number of premature births and the weight of the newborns hospitalised per year was then noted. The incidence of prematurity, the low weights at birth and stillbirths were then compared for the period of lockdown vs. no lockdown. Finally, the profiles of the newborns and their mothers were compared during lockdown (from Tuesday 17th of March to Sunday 10th of May 2020, included) vs. the years 2017–2019 and vs. the date to date period (from 17th of March to the 10th of May for the years 2017, 2018 and 2019). We made comparisons with two different time periods to avoid seasonal variables. The descriptive analysis gave the frequencies and percentages for the qualitative variables as averages and standard deviations. The univariate analyses used the Pearson's chi-square test (or the Fisher's exact test) for qualitative variables and the Anova (or Kruskal-Wallis rank test) for quantitative variables where the mean is presented with range. Statistical analysis used R software (version 4.1.2). All tests were bilateral and a *p*-value <0.05 was considered as statistically significant.

### 2.5. Legal information

This study was referred to the « Comité d'Éthique pour les Recherches Non Interventionnelles ». A favourable response was obtained on the 1st of June 2021, agreement n°2021-034.

## 3. Results

**Figure 2:**
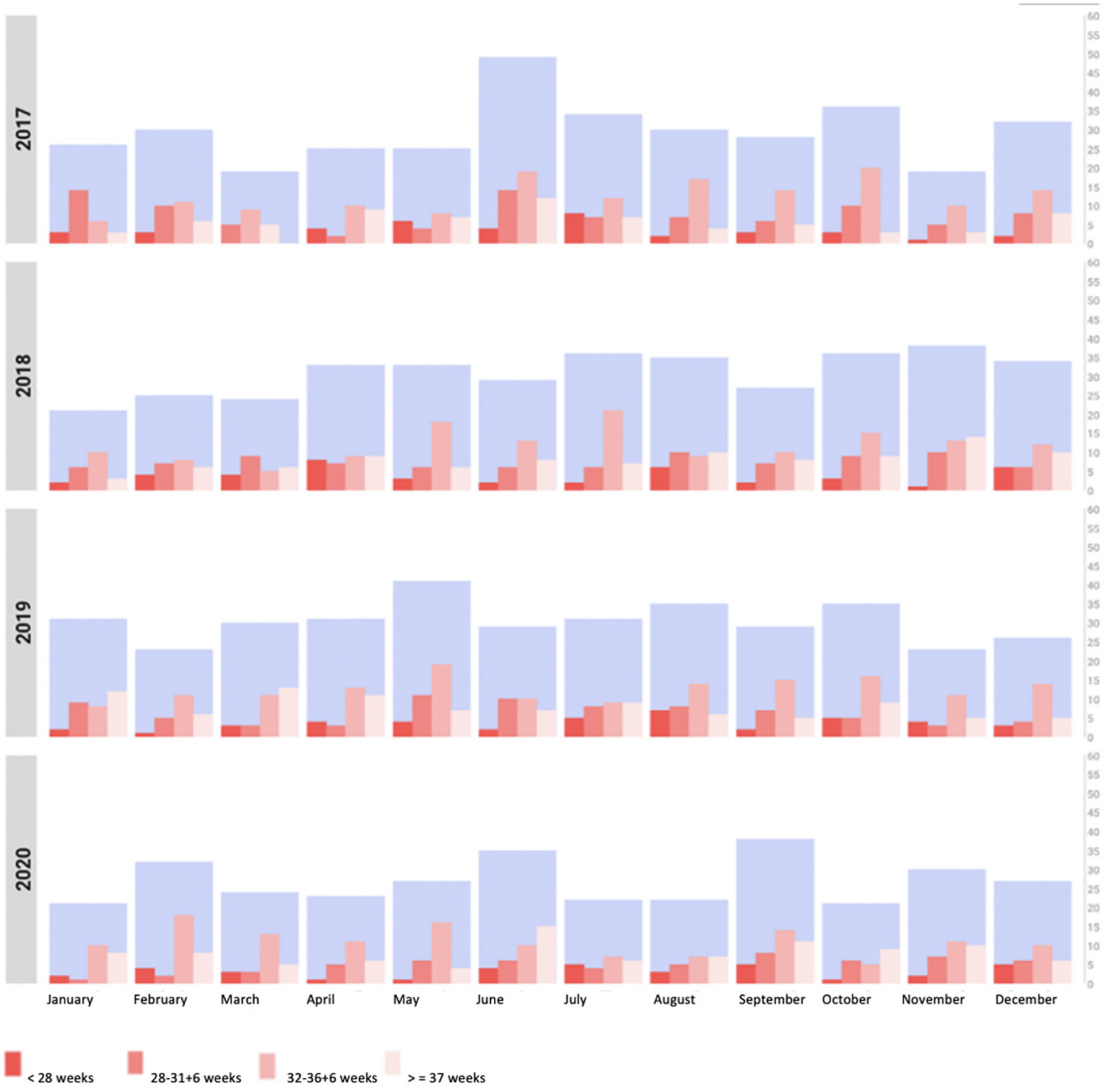
Total births and births according to the term in 2017, 2018, 2019 and 2020.

Despite substantial variation in the number of hospital stays each month, fluctuation was random, a regular seasonal cycles was not observed (Figure 2). The number of premature births <37 weeks of gestation decreased continually each year in a non significant way, giving a lower frequency in 2020 (*N* = 227). We found that this non significant decline was mainly related to « severely premature » newborns (*N* = 59 in 2020 vs. *N* = 92 in 2017, *N* = 89 in 2018, *N* = 76 in 2019). Significant variation in the weights of the newborns was found for the different periods (Table 1). We did not find a significant decrease in either the frequency of prematurity <37 weeks of gestation or low weights at birth during lockdown compared to the average for 2017–2019 (*p* = 0.579 for term pregnancies, *p* = 0.392 for <1,000 g, *p* = 0.698 for <1,500 g) or date to date *p* = 0.689 for term pregnancies, *p* = 0.197 for <1,000 g, and *p* = 0.504 for <1,500 g). There was no significant increase in the number of stillborns during lockdown compared to no lockdown *p* > 0.99) (Table 2). When we compared the profiles of the mothers and newborns according to the term in lockdown vs. no lockdown we noted a significant decrease in the frequency of bronchopulmonary dysplasia of the severely premature, born before 28 weeks of gestation *p* = 0.049) but only when compared to the average for 2017–2019; this significant difference was not found when compared to the date to date period *p* = 0.076). In addition, the profiles of the mothers and newborns were comparable for the different periods (Tables 3, 4).

**Table 1:** Frequency of prematurity in the general population and birth weight of newborns hospitalised in the unit according to the year.

	2017 (*N* = 353)	2018 (*N* = 371)	2019 (*N* = 364)	2020 (*N* = 322)	Total (*N* = 1410)	*p*–value
Term at birth	32.93 (24.00–42.00)	33.30 (24.00–42.00)	33.35 (24.00–42.00)	33.50 (23.00–42.00)	33.27 (23.00–42.00)	0.379
Grouped term at birth						0.143
<28 weeks	39 (11.0%)	43 (11.6%)	42 (11.5%)	36 (11.2%)	160 (11.3%)	
28–31 weeks	92 (26.1%)	89 (24.0%)	76 (20.9%)	59 (18.3%)	316 (22.4%)	
32–36 weeks	150 (42.5%)	143 (38.5%)	151 (41.5%)	132 (41.0%)	576 (40.9%)	
≥37 weeks	72 (20.4%)	96 (25.9%)	95 (26.1%)	95 (29.5%)	358 (25.4%)	
<28 weeks	39 (11.0%)	43 (11.6%)	42 (11.5%)	36 (11.2%)	160 (11.3%)	0.995
<32 weeks	131 (37.1%)	132 (35.6%)	118 (32.4%)	95 (29.5%)	476 (33.8%)	0.156
<37 weeks	281 (79.6%)	275 (74.1%)	269 (73.9%)	227 (70.5%)	1,052 (74.6%)	0.053
Birth weight (g)	1,920 (530.00–5,160.00)	2,030.35 (520.00–5,302.00)	2,006.61 (500.00–4,812.00)	2,062.57 (400.00–4,508.00)	2,004.09 (400.00–5,302.00)	0.209
Birth weight (scale)						0.549
ELBW	42 (11.9%)	42 (11.3%)	49 (13.5%)	45 (14.0%)	178 (12.6%)	
VLBW	82 (23.3%)	76 (20.5%)	76 (20.9%)	52 (16.1%)	286 (20.3%)	
LBW	148 (42.0%)	151 (40.7%)	136 (37.4%)	131 (40.7%)	566 (40.2%)	
NBW	80 (22.7%)	102 (27.5%)	103 (28.3%)	94 (29.2%)	379 (26.9%)	

ELBW, extremely low birth weight <1,000 g; LBW, low birth weight <2,500 g; VLBW, very low birth weight <1,500 g; NBW, normal birth weight >2,500 g.

**Table 2:** Incidence of prematurity, sex, weight and number of stillborns during lockdown (from Tuesday 17th of March to Sunday 10th of May 2020) vs. mean of 2017–2019 and date to date (from the 17th of March to the 10th of May of years 2017, 2018 and 2019).

	Lockdown vs. mean of 2017–2019				Lockdown vs. date to date			
	Lockdown (*N* = 39)	No lockdown (*N* = 1,088)	Total (*N* = 1,127)	*p-*value	Lockdown (*N* = 39)	Lockdown (*N* = 159)	Total (*N* = 198)	*p-*value
Term at birth	33.59 (24.00–40.00)	33.20 (24.00–42.00)	33.21 (24.00–42.00)	0.579	33.59 (24.00–40.00)	33.26 (25.00–42.00)	33.33 (24.00–42.00)	0.689
<28 weeks	2 (5.1%)	124 (11.4%)	126 (11.2%)	0.222	2 (5.1%)	25 (15.7%)	27 (13.6%)	0.084
<32 weeks	12 (30.8%)	381 (35.0%)	393 (34.9%)	0.584	12 (30.8%)	54 (34.0%)	66 (33.3%)	0.705
<37 weeks	30 (76.9%)	825 (75.8%)	855 (75.9%)	0.875	30 (76.9%)	113 (71.1%)	143 (72.2%)	0.465
Sex				0.939				0.704
Boy	22 (56.4%)	95 (55.8%)	117 (55.8%)		22 (56.4%)	95 (59.7%)	117 (59.1%)	
Girl	17 (43.6%)	481 (44.2%)	498 (44.2%)		17 (43.6%)	64 (40.3%)	81 (40.9%)	
Weight <1,000 g	3 (7.7%)	133 (12.2%)	136 (12.1%)	0.392	3 (7.7%)	101 (63.5%)	128 (64.6%)	0.197
Weight <1,500 g	12 (30.8%)	367 (33.8%)	379 (33.7%)	0.698	12 (30.8%)	58 (36.5%)	70 (35.4%)	0.504
				>0.99				>0.99
Nb of total births	452	9,729	10,181		452	1,383	1,835	
Nb of stillborns	9	261	270		9	31	40	

Nb, number.

**Table 3:** Profiles of the mothers and newborns by term during lockdown (from Tuesday 17th of March 2020 to Sunday 10th of May 2020 included) vs. mean of 2017–2019.

	<28 weeks				28–31 ± 6 weeks				32–36 ± 6 weeks			
	Lockdown (*N* = 2)	No lockdown (*N* = 124)	Total (*N* = 126)	*p*-value	Lockdown (*N* = 10)	No lockdown (*N* = 257)	Total (*N* = 267)	*p-*value	Lockdown (*N* = 18)	No lockdown (*N* = 444)	Total (*N* = 462)	*p-*value
Characteristics of mothers and newborns												
Sex				0.782				0.804				0.885
Boy	1 (50%)	74 (59.7%)	75 (59.5%)		6 (60.0%)	144 (56.0%)	150 (56.2%)		10 (55.6%)	239 (53.8%)	249 (53.9%)	
Girl	1 (50%)	50 (40.3%)	51 (40.5%)		4 (40.0%)	113 (44.0%)	117 (43.8%)		8 (44.4%)	205 (46.2%)	213 (46.1%)	
Mother’s age	34 (27.00–41.00)	31.03 (19.00–44.00)	31.08 (19.00–44.00)	0.446	33.30 (25.00–41.00)	31.44 (18.00–56.00)	31.51 (18.00– 56.00)	0.340	33.35 (24.00–44.00)	31.62 (16.00–49.00)	31.68 (16.00– 49.00)	0.232
Premature birth history	0 (0.0%)	12 (9.8%)	12 (9.7.%)	0.641	3 (30.0%)	17 (6.7%)	20 (7.5.%)	0.006	4 (22.2%)	26 (5.9%)	30 (6.5%)	0.007
History of fetal death *in utero*	0 (0.0%)	5 (4.1%)	5 (4.0%)	0.538	0 (0.0%)	7 (2.7%)	7 (2.6%)	0.503	0 (0.0%)	16 (3.6%)	16 (3.5%)	0.090
Complete antenatal corticosteroid therapy	2 (100%)	84 (67.7%)	86 (68.3%)	–	6 (60.0%)	205 (80.1%)	211 (73.9%)	0.270	9 (50.0%)	259 (58.3%)	268 (58.0%)	0.745
Magnesium sulfate	1 (50.0%)	104 (83.9%)	105 (83.3%)	0.202	10 (100.0%)	208 (81.2%)	218 (82.0%)	0.130	4 (22.2%)	72 (16.2%)	76 (16.5%)	0.503
Group B Streptococcus testing				0.642				0.681				0.845
Negative	2 (100.0%)	85 (69.1%)	87 (69.6%)		7 (70.0%)	157 (61.1%)	164 (61.4%)		12 (66.7%)	305 (68.7%)	317 (68.6%)	
Positive	0 (0.0%)	14 (11.4%)	14 (11.2%)		0 (0.0%)	16 (6.2%)	16 (6.0%)		1 (5.6%)	35 (7.8%)	36 (7.8%)	
Not done	0 (0.0%)	24 (19.5%)	24 (19.2%)		3 (30.0%)	84 (32.7%)	87 (32.6%)		5 (27.8%)	100 (22.5%)	105 (22.7%)	
Perinatal context				0.857				0.545				0.766
Threat of premature labor	1 (50.0%)	59 (47.6%)	60 (47.6%)		4 (40.0%)	64 (24.9%)	68 (25.5%)		5 (27.8%)	97 (21.8%)	102 (22.1%)	
PPROM	0 (0.0%)	25 (20.2%)	25 (19.8%)		0 (0.0%)	45 (17.5%)	45 (16.9%)		5 (27.8%)	135 (30.4%)	140 (30.3%)	
Pre-eclampsia/vascular	0 (0.0%)	13 (10.5%)	13 (10.3%)		4 (40.0%)	72 (28.0%)	76 (28.5%)		7 (38.9%)	132 (29.7%)	139 (30.1%)	
Metrorrhagia	0 (0.0%)	4 (3.2%)	4 (3.2%)		0 (0.0%)	9 (3.5%)	9 (3.4%)		0 (0.0%)	18 (4.1%)	18 (3.9%)	
Chorio amnionitis	1 (50.0%)	21 (16.9%)	22 (17.5%)		2 (20.0%)	52 (20.2%)	54 (20.2%)		0 (0.0%)	16 (3.6%)	16 (3.5%)	
Other	0 (0.0%)	2 (1.6%)	2 (1.6%)		0 (0.0%)	15 (5.8%)	15 (5.6%)		1 (5.6%)	46 (10.4%)	47 (10.2%)	
Delivery route				0.873				0.126				0.581
Vaginal	1 (50.0%)	69 (55.6%)	70 (55.6%)		6 (60.0%)	93 (36.2%)	99 (37.1%)		7 (38.9%)	202 (45.5%)	209 (45.2%)	
Caesarean section	1 (50.0%)	55 (44.4%)	56 (44.4%)		4 (40.0%)	164 (63.8%)	168 (62.9%)		11 (61.1%)	242 (54.5%)	253 (54.8%)	
Perinatal outcomes												
Respiratory distress syndrome	2 (100.0%)	124 (100.0%)	126 (100.0%)	–	10 (100.0%)	240 (93.4%)	250 (93.6%)	0.401	8 (44.4%)	198 (44.6%)	206 (44.6%)	0.990
Surfactant	2 (100.0%)	85 (68.5%)	87 (69.0%)	0.340	5 (50.0%)	59 (23.0%)	64 (24.0%)	0.049	0 (0.0%)	17 (3.8%)	17 (3.7%)	0.398
Bronchopulmonary dysplasia	0 (0.0%)	82 (66.7%)	82 (65.6%)	0.049	3 (30.0%)	52 (20.2%)	55 (20.6%)	0.454	0 (0.0%)	2 (0.5%)	2 (0.4%)	0.775
Intraventicular hemorrhage	1 (50.0%)	51 (41.1%)	52 (41.3%)	0.800	1 (10.0%)	37 (14.4%)	38 (14.2%)	0.696	1 (5.6%)	38 (8.6%)	39 (8.4%)	0.651
Periventricular leukomalacia	0 (0.0%)	4 (3.2%)	4 (3.2%)	0.497	1 (10.0%)	4 (1.6%)	5 (1.9%)	0.142	0 (0.0%)	2 (0.5%)	2 (0.4%)	0.069
Necrotizing enterocolitis	0 (0.0%)	13 (10.5%)	13 (10.3%)	0.629	0 (0.0%)	6 (2.3%)	6 (2.2%)	0.625	0 (0.0%)	5 (1.1%)	5 (1.1%)	0.651
Neonatal infection	0 (0.0%)	40 (32.3%)	40 (31.7%)	0.591	0 (0.0%)	32 (12.5%)	32 (12.0%)	0.312	0 (0.0%)	12 (2.7%)	12 (2.6%)	0.413
Retinopathy	0 (0.0%)	6 (4.8%)	6 (4.8%)	0.909	0 (0.0%)	0 (0.0%)	0 (0.0%)	–	0 (0.0%)	0 (0.0%)	0 (0.0%)	–
Ductus arteriosus	0 (0.0%)	67 (54.0%)	67 (54.0%)	0.122	1 (10.0%)	20 (7.8%)	21 (7.9%)	0.798	2 (11.1%)	5 (1.1%)	7 (1.5%)	<0.001
Death	1 (50.0%)	34 (27.4%)	35 (27.8%)	0.479	2 (20.0%)	13 (5.1%)	15 (5.6%)	0.044	1 (5.6%)	10 (2.3%)	11 (2.4%)	0.367

PPROM, preterm premature rupture of the membranes.

*p* value < 0.05 was consider statistically significant.

**Table 4:** Profiles of the mothers and newborns by term during lockdown (from Tuesday 17th of March 2020 to Sunday 10th of May 2020) vs. date to date (from the 17th of March to the 10th of May of years 2017, 2018 and 2019).

	<28 weeks				28–31 ± 6 weeks				32–36 ± 6 weeks			
	Lockdown (*N* = 2)	No lockdown (*N* = 25)	Total (*N* = 27)	*p-*value	Lockdown (*N* = 10)	No lockdown (*N* = 29)	Total (*N* = 39)	*p-*value	Lockdown (*N* = 18)	No lockdown (*N* = 59)	Total (*N* = 77)	*p-*value
Characteristics of mothers and newborns												
Sex				0.957				0.754				0.922
Boy	1 (50%)	13 (52.0%)	14 (51.9%)		6 (60.0%)	19 (65.5%)	25 (64.1%)		10 (55.6%)	32 (54.2%)	42 (54.5%)	
Girl	1 (50%)	12 (48.0%)	13 (48.1%)		4 (40.0%)	10 (34.5%)	14 (35.9%)		8 (44.4%)	27 (45.8%)	35 (45.5%)	
Mother’s age	34 (27.00–41.00)	31.80 (21.00–41.00)	31.96 (21.00–41.00)	0.654	33.30 (25.00–41.00)	29.59 (23.00–38.00)	30.54 (23.00–41.00)	0.037	33.35 (24.00–44.00)	32.44 (19.00–43.00)	32.65 (19.00–44.00)	0.566
Premature birth history	0 (0.0%)	2 (8.0%)	2 (7.4.%)	0.678	3 (30.0%)	1 (3.4%)	4 (10.3.%)	0.017	4 (22.2%)	6 (10.1%)	10 (13.0%)	0.203
History of fetal death *in utero*	0 (0.0%)	0 (0.0%)	0 (0.0%)	–	0 (0.0%)	1 (3.4%)	1 (2.6%)	–	0 (0.0%)	1 (1.7%)	1 (1.3%)	0.656
Complete antenatal corticosteroid therapy	2 (100%)	16 (64.0%)	18 (66.7%)	–	6 (60.0%)	20 (69.0%)	26 (66.7%)	0.868	9 (50.0%)	38 (64.4%)	47 (61.0%)	0.441
Magnesium sulfate	1 (50.0%)	18 (72.0%)	19 (70.4%)	0.512	10 (100.0%)	23 (79.3%)	33 (84.6%)	0.118	4 (22.2%)	12 (20.3%)	16 (20.8%)	0.863
Group B Streptococcus testing				0.618				0.419				0.926
Negative	2 (100.0%)	16 (64.0%)	18 (66.7%)		7 (70.0%)	14 (48.3%)	21 (53.8%)		12 (66.7%)	34 (57.6%)	46 (59.8%)	
Positive	0 (0.0%)	4 (16.0%)	4 (16.0%)		0 (0.0%)	2 (6.9%)	2 (5.1%)		1 (5.6%)	4 (6.7%)	5 (6.5%)	
Not done	0 (0.0%)	4 (16.0%)	4 (16.0%)		3 (30.0%)	13 (44.8%)	16 (41.0%)		5 (27.8%)	17 (28.8%)	22 (28.6%)	
Perinatal context				–				0.588				0.184
Threat of premature labor	1 (50.0%)	13 (52.0%)	14 (51.9%)		4 (40.0%)	10 (34.5%)	14 (35.9%)		5 (27.8%)	4 (6.8%)	9 (11.7%)	
PPROM	0 (0.0%)	1 (4.0%)	1 (3.7%)		0 (0.0%)	4 (13.8%)	4 (10.3%)		5 (27.8%)	24 (40.7%)	29 (37.7%)	
Pre-eclampsia/vascular	0 (0.0%)	2 (8.0%)	2 (7.4%)		4 (40.0%)	8 (27.6%)	12 (30.8%)		7 (38.9%)	21 (35.6%)	28 (36.4%)	
Metrorrhagia	0 (0.0%)	1 (4.0%)	1 (3.7%)		0 (0.0%)	3 (10.3%)	3 (7.7%)		0 (0.0%)	2 (3.4%)	2 (2.6%)	
Chorio amnionitis	1 (50.0%)	8 (32.0%)	9 (33.3%)		2 (20.0%)	3 (10.3%)	5 (12.8%)		0 (0.0%)	3 (5.1%)	3 (3.9%)	
Other	0 (0.0%)	0 (0.0%)	0 (0.0%)		0 (0.0%)	1 (3.4%)	1 (2.6%)		1 (5.6%)	5 (8.5%)	6 (7.8%)	
Delivery route				0.869				0.065				0.994
Vaginal	1 (50.0%)	14 (56.0%)	15 (55.6%)		6 (60.0%)	8 (27.6%)	14 (35.9%)		7 (38.9%)	23 (39.0%)	30 (39.0%)	
Caesarean section	1 (50.0%)	11 (44.0%)	12 (44.4%)		4 (40.0%)	21 (72.4%)	25 (64.1%)		11 (61.1%)	36 (61.0%)	47 (61.0%)	
Perinatal outcomes												
Respiratory distress syndrome	2 (100.0%)	25 (100.0%)	27 (100.0%)	–	10 (100.0%)	27 (93.1%)	37 (94.9%)	0.394	8 (44.4%)	23 (39.0%)	31 (40.3%)	0.679
Surfactant	2 (100.0%)	17 (68.0%)	19 (70.4%)	0.340	5 (50.0%)	8 (27.6%)	13 (33.3%)	0.195	0 (0.0%)	1 (1.7%)	1 (1.3%)	0.578
Bronchopulmonary dysplasia	0 (0.0%)	16 (64.0%)	16 (59.3%)	0.076	3 (30.0%)	5 (17.2%)	8 (20.5%)	0.968	0 (0.0%)	0 (0.0%)	0 (0.0%)	–
Intraventicular hemorrhage	1 (50.0%)	10 (40.0%)	11 (40.7%)	0.782	1 (10.0%)	5 (17.2%)	6 (15.4%)	0.584	1 (5.6%)	5 (8.5%)	6 (7.8%)	0.686
Periventricular leukomalacia	0 (0.0%)	0 (0.0%)	0 (0.0%)	–	1 (10.0%)	1 (3.4%)	2 (5.1%)	0.331	0 (0.0%)	0 (0.0%)	0 (0.0%)	–
Necrotizing enterocolitis	0 (0.0%)	3 (12.0%)	3 (11.1%)	0.603	0 (0.0%)	0 (0.0%)	0 (0.0%)	–	0 (0.0%)	0 (0.0%)	0 (0.0%)	–
Neonatal infection	0 (0.0%)	10 (40.0%)	10 (37.0%)	0.501	0 (0.0%)	2 (6.9%)	2 (5.1%)	0.395	0 (0.0%)	1 (1.7%)	1 (1.3%)	0.576
Retinopathy	0 (0.0%)	0 (0.0%)	0 (0.0%)	–	0 (0.0%)	0 (0.0%)	0 (0.0%)	–	0 (0.0%)	0 (0.0%)	0 (0.0%)	–
Ductus arteriosus	0 (0.0%)	14 (56.0%)	14 (51.9%)	0.127	1 (10.0%)	1 (3.4%)	2 (5.1%)	0.418	2 (11.1%)	1 (1.7%)	3 (3.9%)	0.071
Death	1 (50.0%)	8 (32.0%	9 (33.3%)	0.603	2 (20.0%)	1 (3.4%)	3 (7.7%)	0.090	1 (5.6%)	0 (0.0%)	1 (1.3%)	0.068

PPROM, preterm premature rupture of the membranes.

*p* value < 0.05 was consider statistically significant.

## 4. Discussion

This study did not reveal any evidence of a significant decrease in either prematurity or low birth weights during lockdown. There was no significant increase in the number of stillbirths. There was no significant difference in the profiles of the mothers or newborns. The collection of information is limited by the retrospective nature of the study since some of the data was missing or absent, which leads to a degree of bias regarding the information. Some of the absent information concerned maternal risk factors such as the preconception body mass index, the marital status, the economic status and level of education. In addition, the study lacked power due to the low number of individuals included, despite the collection of date over four years. Thus, the monocentric nature of the study does not allow generalisation of the results, which limits the external validity. However, the strength of the study lies in the exhaustive collection of hospitalised births admitted into the critical care neonatal unit and neonatology department of the Nice University Hospital during lockdown as well as the uniformity of the data and the collection of the number of stillborns. The number of stillborns was not different between periods and did not have an impact on prematurity.

The results are in agreement with some of the published literature and in particular with some meta-analyses concerning this subject, including the meta-analyses of Vaccaro et al. (5), Chmielewska et al. (6), and Yang et al. (7). Several studies showed that lockdown did not result in a decrease in prematurity, including in France (8), Spain (9), Sweden (10), Israel (11), the United States of America (12), the United Kingdom (13), and China (14). A meta-analysis by Vaccaro *et al*. evaluated the impact of lockdown on prematurity, low birth weight <2,500 g and stillbirths but did not report an association between lockdown and these issues (5). Another meta-analysis by Chmielewska et al. did not find an association between lockdown and prematurity and low birth weight <2,500 g (6). A third meta-analysis by Yang et al. did not find any association between prematurity and lockdown in studies using regional/national data (7). In contrast, the initial published results were in favour of a decrease in prematurity and a low weight at birth during lockdown, notably by studies performed in spring of 2020 in Ireland (15) and Denmark (16). Other studies evaluating the association between prematurity, low birth weight and the number of stillborns performed around the world also found similar results, including in Iran (17), Australia (18), Saudi Arabia (19), Italy (20), Netherlands (21) and Austria (22). Two meta-analyses also demonstrated a decrease of prematurity during lockdown. Calvert et al. showed small reductions in preterm birth in high income and upper middle income countries during the first, second and third months of lockdown (but not in the fourth month) (23). Yao et al. identified a reduction in preterm birth during pandemic compared with pre pandemic period, but further subgroup analysis showed that there were no difference in studies from multicenter or low and middle income countries (24). According to these studies the potential positive effects of lockdown on prematurity and low birth weight were due to several factors. These included the decrease in social interaction, the shutdown of schools, the waring of masks and an increase in hygiene, which may have decreased the risk of contact with pathogens and thus a decrease in maternal infection (15–21).

There are many factors of risk of prematurity but the ethology is sometimes not well understood. They can be maternal, obstetric, infectious or environmental. The factors of risk of prematurity that may have changed during lockdown include atmospheric pollution (due to limited journeys), rest and maternal stress (with more time at home, the set up of work from home, more family support with the partner at home and certain financial assistance from governments). However, these factors of risk remain debatable and have yet to be proven. Atmospheric pollution, which may have decreased during lockdown, is a controversial factor of risk since it depends on the type of particule studied and the trimester of exposure (25, 26). With respect to rest, a systematic review by Cochrane (27) published in 2015, did not find evidence to show that bedrest reduced prematurity, on the contrary it may have negative effects such as an increase in demineralisation of bone and deconditioning during exercise or an increased risk of deep vein thrombosis (28). With respect to maternal stress, this is a subjective element with different definitions according to the studies so it is difficult to establish an association with perinatal issues such as prematurity. While it is qualified as a risk factor some studies do not report any difference and even report a decrease in prematurity (29). There also exists a hypothesis suggesting that stressful events do not have the same impact when experienced in the first, second or third trimester of pregnancy, with a higher degree of stress at the beginning of pregnancy (30).

It should be noted that preventive measures exist to reduce prematurity (31, 32). There are three types: primary prevention that concerns all women, secondary prevention to reduce and eliminate already existing risks and tertiary prevention to improve the outcome of infants born prematurely. Tertiary prevention has been the most developed in recent years, with the set up of networks of organisations providing perinatal care and health care to mothers and their newborns in adapted maternities, of antenatal corticosteroid therapy use and administration of magnesium sulphate.

## 5. Conclusion

The objective of our study was to examine the consequences of lockdown in France due to the SARS-CoV-2 pandemic on prematurity before 37 weeks of gestation in the level III maternity of the Nice University Hospital. We did not find evidence of an association between lockdown and prematurity, which is in agreement with published meta-analyses. Certain factors of risk such as atmospheric pollution, rest and maternal stress that were discussed during lockdown are debatable and their involvement remains to be demonstrated. The identification of the factors of risk of prematurity and the preventive measures are still a major public health issue.

## Data Availability

The raw data supporting the conclusions of this article will be made available by the authors, without undue reservation.
